# Dissections of Larval, Pupal and Adult Butterfly Brains for Immunostaining and Molecular Analysis

**DOI:** 10.3390/mps4030053

**Published:** 2021-08-05

**Authors:** Yi Peng Toh, Emilie Dion, Antónia Monteiro

**Affiliations:** 1 Department of Biological Sciences, National University of Singapore, 14 Science Drive 4, Singapore 117543, Singapore; toh.yi.peng.2014@vjc.sg (Y.P.T.); antonia.monteiro@nus.edu.sg (A.M.); 2 Yale-NUS College, 10 College Avenue West, Singapore 138609, Singapore

**Keywords:** brain dissections, butterflies, caterpillars, pupa, *Bicyclus anynana*

## Abstract

Butterflies possess impressive cognitive abilities, and investigations into the neural mechanisms underlying these abilities are increasingly being conducted. Exploring butterfly neurobiology may require the isolation of larval, pupal, and/or adult brains for further molecular and histological experiments. This procedure has been largely described in the fruit fly, but a detailed description of butterfly brain dissections is still lacking. Here, we provide a detailed written and video protocol for the removal of *Bicyclus anynana* adult, pupal, and larval brains. This species is gradually becoming a popular model because it uses a large set of sensory modalities, displays plastic and hormonally controlled courtship behaviour, and learns visual mate preference and olfactory preferences that can be passed on to its offspring. The extracted brain can be used for downstream analyses, such as immunostaining, DNA or RNA extraction, and the procedure can be easily adapted to other lepidopteran species and life stages.

## 1. Introduction

Despite having small brains, insects’ cognitive abilities are impressive. They can process a large set of information from their environment and adjust their behaviour accordingly [1]. Many species possess remarkable learning and memorisation skills [2,3]. Examples of sophisticated behaviours include concept and category learning in honeybees [4], tool use in ants [5], facial pattern recognition in wasps [6,7], and counting ability in bees [8,9].

The neural mechanisms responsible for these sophisticated behaviours have interested researchers for decades (e.g., [10,11,12]). These mechanisms were largely investigated in the fruit fly (e.g., [13,14,15,16]), honey bee (e.g., [17,18,19]), and more recently in other insects, such as crickets (e.g., [20,21,22]), mosquitos (e.g., [23]), or cockroaches (e.g., [24,25]), among others. Recently, studies on butterflies have also increased, motivated by the large diversity of behaviours that they can perform.

Butterflies are becoming a popular system in the fields of evolutionary neurobiology and neuroethology because they use a large set of sensory modalities for their survival and reproduction [26]. They process visual [27], olfactory [28], auditory [29], and gustatory [30] signals to help them forage, search for a host plant, select mates, avoid predators, or migrate. They can also memorise information from their environment and adjust their behaviour accordingly (e.g., [31,32]). Butterflies’ visual and olfactory systems are also crucial mediators of reproductive isolation and speciation [33,34].

The growing exploration of the butterflies’ neurobiology prompted the development of experimental techniques for producing relevant molecular and neuroanatomical data. Brain dissections are an essential step in the experimental process (e.g., [35,36,37]). Protocols describing the dissection of *Drosophila* brains are currently available [38,39,40,41,42], but a detailed description of butterfly brain dissections at various developmental stages is still lacking.

Here, we describe the process of larval, pupal, and adult butterfly brain removal using a video. We also provide a complete written description of the processes, with a list of necessary tools and chemicals. The dissection methods can be applied to different butterfly species, and the brains can be used for DNA or RNA extraction, or for immunostaining. We record the dissection process in the Bush brown butterfly *Bicyclus anynana*, which is especially interesting due to its plastic and sex-role reversed courtship behaviour [43,44,45,46]. Individuals are also capable of learning visual and olfactory cues [47,48,49], and they can transmit the learned odour preferences to their offspring [48,49]. Because the molecular basis of these unique behaviours can begin to be examined at the level of the brain, it is important to extract this organ for downstream analyses.

We dissected the brains of two-day-old butterflies, those of pupae at 30% development, and those of 5th instar larvae. These specific ages were chosen because *B. anynana* adult individuals showed a change in sex-pheromone and wing-pattern preferences on day two after exposure to the newly emerged females [47,48]. The 30% pupal development corresponds to the critical stage where variation in hormone levels influences adult courtship behaviour [50,51]. We chose to dissect 5th instar larval brains because they are bigger at this stage, and they provide higher amounts of RNA and DNA. However, *B. anynana* larvae start preferring novel food odours from the 3rd instar, and keep the same preference until pupation [49]. To show that our methods can be easily applied to other families and individuals of various sizes, we also dissected the brains of adults, pupae, and caterpillars of *Pieris canidia* (family: Pieridae) at the same stages as *B. anynana*, and adults of two additional species from the Nymphalidae and Papilionidae.

## 2. Experimental Design

**Figure 1:**
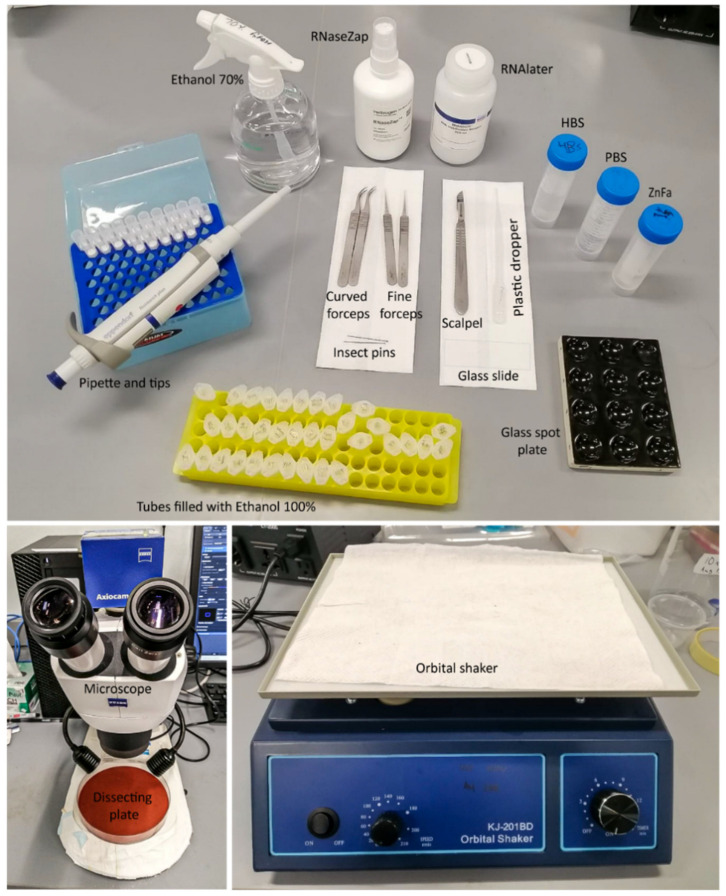
Equipment and materials needed for the dissection of adult, pupal, and larval brains.

**Figure 2:**
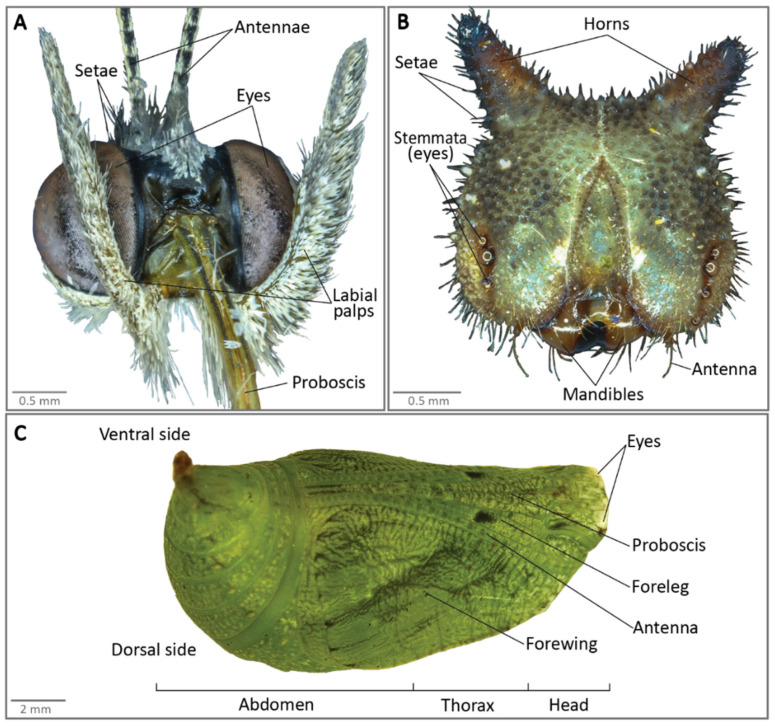
Anatomy of (**A**) a *B. anynana* butterfly head (front view), (**B**) a caterpillar head (front view), and (**C**) of a pupa.

All materials and equipment needed for brain dissections are described in Figure 1. Figure 2 presents the anatomy of a larval head, adult head and of a pupa.

### 2.1. Materials

- Curved forceps (Dumont; Dumont Switzerland, Montignez, Switzerland; Cat. No.: 11274-20);
- Superfine dissecting forceps (Dumont; Dumont Switzerland, Montignez, Switzerland; Cat. No.: 82027-402);
- Blade holder (Swann-Morton No. 4; Swann-Morton, Sheffield, UK; Cat. No.: 0934);
- Surgical blades (Swann-Morton No. 4; Swann-Morton, Sheffield, UK; Cat. No.: 0115);
- Two dissection silicone plates (Dragon Skin 30 Mould Making Silicone Rubber; Cat. No.: 0751635278417, Petri plate; Sigma-Aldrich; Sigma-Aldrich, Singapore; Cat. No.: P5981-100EA). Use black or red silicon to best see the white brains, or place a black background underneath a transparent silicon plate;
- Glass spot plate (PYREX^TM^; Corning, Corning, NY, USA; Cat. No.: 722085);
- Insect pins (BioQuip; BioQuip, Rancho Dominguez, CA, USA; Cat. No.: 1208B2);
- Glass slides (Biomedia, Singapore, Cat. No.: BMH.880102);
- Plastic pipettes (unbranded, Singapore), 1000 µL;
- Filter tips (Maximum Recovery, Axygen, Union City, CA, USA; no. TF-1000-L-R-S);
- Microcentrifuge tubes 1.5 mL (Eppendorf, Hamburg, Germany; Cat. no.: T9661-500EA).

### 2.2. Equipment

- Zeiss Dissection Microscope (Carl-Zeiss, Jena, Germany; Stemi 305);
- Orbital Shaker (Kangjian, Jiangyan City, China).

### 2.3. Reagents and Solutions

- NaCl (Sigma-Aldrich; Sigma-Aldrich, Singapore; Cat. No.: S9888-500G);
- KCl (Sigma-Aldrich; Sigma-Aldrich, Singapore; Cat. No.: P9541-500G);
- CaCl_2_ (Sigma-Aldrich; Sigma-Aldrich, Singapore; Cat. No.: C1016-100G);
- ZnCl2 (Sigma-Aldrich; Sigma-Aldrich, Singapore; Cat. No.: 208086-100G);
- K_2_HPO_4_ (Sigma-Aldrich; Sigma-Aldrich, Singapore; Cat. No.: P3786-500G);
- Sucrose (Sigma-Aldrich; Sigma-Aldrich, Singapore; Cat. No.: S0389-1KG);
- HEPES (Sigma-Aldrich; Sigma-Aldrich, Singapore; Cat. No.: H3375-100G);
- 16% Formaldehyde (ThermoFisher Scientific, Singapore; Cat. No.: 28908);
- Ethanol absolute (ThermoFisher Scientific, Singapore, Cat. No.: 64175);
- RNALater^®^ RNA Stabilisation Reagent (Qiagen, Singapore, Cat. No.: 76104);
- RNAse*Zap*^TM^ RNase Decontamination Solution (ThermoFisher Scientific, Singapore, Cat. No.: AM9780);

## 3. Dissection of Adult Brains

### 3.1. Preparation for Dissection

- Freeze anaesthetise the butterflies in a −20 °C freezer for 3–4 min;
- Wash the dissection tools and plates in 70% ethanol prior to dissection;
- Place a few drops of HEPES-buffered saline (HBS) onto a dissection plate using a plastic pipette, and set it aside (see recipe for HBS and all other reagents at the end of this article);
- If the brain tissues need to undergo immunostaining, fill a well in the spot plate with ZnFA solution using a plastic pipette, and set it aside before starting any dissection. If the brains are used for DNA or RNA extraction, fill the RNAse-free Eppendorf tubes with about 300 µL of 100% ethanol or RNAlater, respectively. Place only up to 3 brains in the same well (for immunostaining). Number of brains to be pooled in one tube varies with the amount of DNA or RNA needed for downstream analyses;

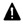
**CRITICAL STEP** If DNA or RNA work is involved, it is recommended to wipe the equipment with RNAseZap in step 2, and use a micropipette and filter tips instead in steps 3 and 4.

### 3.2. Dissection Procedure 

#### 3.2.1. Exposure of Adult Brains

**Figure 3:**
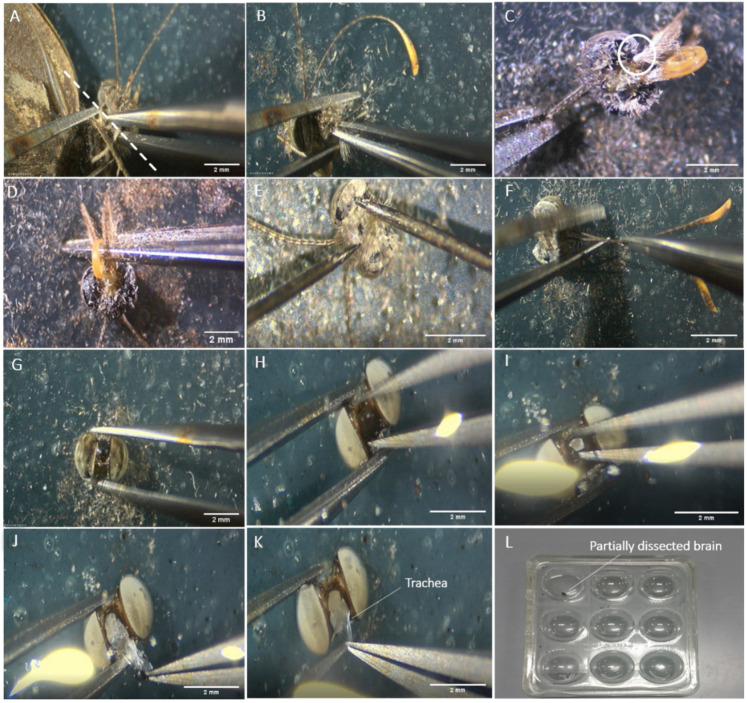
Exposure of an adult brain and fixation. Steps in (**A**–**G**) are performed dry while (**H**–**L**) are submerged in solution. (**A**) The ideal point of pull when detaching the head of the adult from the rest of the body is indicated by the white dashed line. (**B**) The remaining thoracic regions attached to the butterfly head are grabbed with fine forceps and pulled away. (**C**) The thoracic region is removed until the proximal ends of the palps are seen (circled in white). (**D**) The labial palps and the proboscis are then pulled away from the head. € The setae attached to the front and back of the cuticle are scraped off gently using insect pins, to prevent setae from clouding the surface of the dissecting solution in the later steps. (**F**) The antennae are subsequently removed. (**G**) The head is picked up gently using curved forceps and transferred to a dissecting plate with HBS. (**H**) The head is secured with curved forceps to allow easier access to the cuticle in between the two eyes (frontal view). (**I**) A small tear is made in the piece of cuticle in between the two eyes. (**J**) The brain is exposed with the region of the cuticle in between the two eyes removed, subsequently exposing the trachea underneath. (**K**) The sticky white trachea just below the surface of the cuticle are removed. (**L**) The exposed brain is submerged in a ZnFA solution in a spot plate well, and the opening is covered with a glass slide to prevent evaporation of the solution and drying of the tissues. The glass plate is subsequently placed on an orbital shaker with a setting of 80–100 rmp for 16–20 h.

- Take the butterfly out of the freezer and place it on a second clean dissection plate. The first series of steps are performed dry. Detach the head of the butterfly from the rest of the body using a pair of superfine dissecting forceps. Pull the head away from the body using your dominant hand while pressing down on the wings with the other hand. Ensure the point of pull is as close to the head as possible, to reduce the amount of thoracic tissue needed to be removed (Figure 3A);
- Remove the rest of the butterfly from the dissecting plate, leaving only the head. There will be some remaining thoracic regions left on the back of the head most of the time. Secure the head from moving with the forceps in your non-dominant hand, while pulling away the thoracic region with the other forceps in your dominant hand (Figure 2B). Remove the thoracic tissue until you can visualise the connection point of the labial palps to the head (Figure 2A and Figure 3C);
- Detach the labial palps and proboscis (Figure 2A and Figure 3C,D) by pulling them hard away from the head. The easiest and fastest way to completely remove the palps and the proboscis is to grip them from the back of the head and pull them away from the head using the forceps in your dominant hand (Figure 3D), while gently pressing the part of the head where the proximal ends of the labial palps are located, using the other forceps in your non-dominant hand;
- Using a pair of insect pins, gently scrape off the setae that are attached to the cuticle surrounding the head. Gently press the head down with one insect pin while scraping the setae off with another (Figure 2A and Figure 3E);

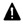
**CRITICAL STEP** Be careful not to apply too much pressure when scraping near the eye, as a slight increase in pressure will damage the optic lobe. This step will reduce the number of setae floating on the dissecting solution, and allow for easier exposure of the brain tissue in the later steps;

- Detach the antennae (Figure 2A and Figure 3F) using a pair of curved forceps. Pull the antennae hard away from the head using the forceps in your dominant hand, while gently pressing down, to the extent of just touching the head with the other forceps in your non-dominant hand (Figure 3F);
- Gently pick up the head using a curved forceps and transfer it into the previously prepared dissection plate with a few drops of HBS (enough to cover the tissue fully, about 180 µL here) for subsequent exposure of the brain tissue (Figure 3G);
- Secure the head between the curved forceps and fully submerge it into HBS before exposing the tissue;

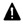
**CRITICAL STEP** The head will tend to float on the surface of the saline solution at first. It is critical to ensure it is fully submerged (by securing the head between the ends of the forceps and then sinking it in the plate so that the saline solution fully covers the head). This ensures that the brain will be exposed within the solution and not outside of it, reducing tissue damage;

- Secure the head with the curved forceps facing upwards, to get a grip on the region of the cuticle in between the two eyes (Figure 3H);
- Make a small tear in the cuticle between the eyes (Figure 2I) and use it as an opening point to remove the rest of the cuticle (Figure 3J);
- Pull out any easily accessible trachea, using curved forceps in your dominant hand (Figure 3K).

Note: If the brains are used for DNA and RNA extraction, skip Section 3.2.2 (Fixation), and continue the dissection procedure directly as described in Section 3.2.3 below.

#### 3.2.2. Fixation of Adult Brains for Immunostaining

**Figure 4:**
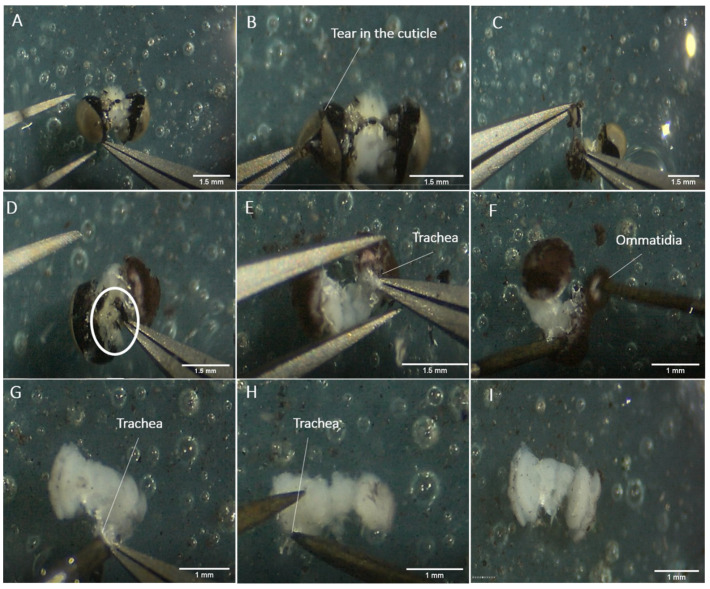
Dissection of an adult brain. (**A**) A small part of the brain is grabbed to submerge it fully in HBS. (**B**) While the brain is submerged, a superficial tear in the cuticle of one eye is made. (**C**) A small piece of cuticle is removed on the side of the eye. (**D**) The non-brain tissue that is loosely attached to the back of the brain is removed, as indicated by the white circle. (**E**) The cuticle of both eyes is removed, exposing some trachea. The trachea is the white, stringy, and sticky substance attached to the brain tissues. It is important to control the pulling force so that the brain tissues are not accidentally teared. (**F**) The ommatidia are packed together into a reddish-brown spongy tissue, and this is removed from one side of the eye. (**G**) Some trachea still attached is pulled away with curved forceps. (**H**) The remaining trachea can also be scraped away with insect pins. (**I**) The fully dissected brain can be used for further analyses.

- Gently lift the head up with the exposed brain with the curved forceps and transfer it into a well filled with ZnFA solution (Figure 3L). Cover the well with a glass slide, then place the well plate on an orbital shaker and agitate it for 16–20 h at 80–100 rates per minute (rmp);
- After fixing overnight, transfer each fixed brain from the well into a dissection plate with a few drops of HBS by gently lifting it up using the curved forceps;
- Again, the brain might float on the surface of the solution. Grab a small part of the brain with the curved forceps and fully submerge it into the solution (Figure 4A).

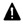
**CRITICAL STEP** Ensure to cover the well with the glass slide so that the fixing solution does not evaporate overnight.

#### 3.2.3. Dissection of the Adult Brain

- Gently make a superficial tear in the cuticle of one eye (Figure 4B);
- Then start breaking off the cuticle into small pieces and remove it gently (Figure 4C);

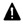
**CRITICAL STEP** Do not pull strongly, or the whole optic lobe will be detached from the rest of the brain. If the cuticle cannot be broken off, try to dislodge it via a rotating pull. The region of the cuticle nearest to the central brain sticks very closely to the brain tissues, and is hard to remove via normal pulling without damaging the tissues. The easiest way to remove it will be to pull it along the orientation of the eye, going from the dorsal to the ventral point in a circular motion, and breaking it off naturally;

- After clearing out the cuticle of one eye, remove the piece of non-brain tissue that is loosely attached to the back of the brain, with small bits of cuticle on it (Figure 4D);
- Proceed to remove the cuticle of the other eye by repeating steps 3 and 4;
- Remove the easily accessible trachea (Figure 4E);
- Then, scrape away the main body of the ommatidia and the basement membrane of the retina from the optic lobes, using a pair of insect pins (Figure 4F). These tissues are darkly coloured;

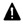
**CRITICAL STEP** Do not scrape too hard as the basement membrane is firmly attached to the lamina of the optic lobe. The lamina gets easily destroyed with just a little pulling pressure;

- Remove any remaining trachea by pulling gently with a curved forceps (Figure 4G), while pressing the brain down with an insect pin or by using insect pins to scrape them off the brain tissue (Figure 4H);
- Transfer the fully dissected brain back to the well for downstream immunostaining using a pipette (Figure 3I), or into an Eppendorf tube filled with RNAlater (for RNA extraction) or 100% ethanol (for DNA extraction).

## 4. Dissection of Pupal Brains

### 4.1. Preparation for Dissection

- Place the pupae on ice for about 15 min prior to dissection;
- Wash the forceps, scalpel, and dissection plate with 70% ethanol. If the brains are used for RNA extraction, use RNAzap instead;
- Prepare RNase-free Eppendorf tubes or well plates filled with the solution appropriate for the subsequent experiment, and place them close to the dissection microscope. For DNA and RNA extraction, prepare tubes with about 300 µL of 100% ethanol and RNAlater, respectively. For immunostaining experiments, place about 500 µL of the ZnFa fixative solution in the wells. Place a drop of cold PBS on the dissection plate (enough to fully cover the tissue, here about 500 µL).

### 4.2. Dissection Procedure

**Figure 5:**
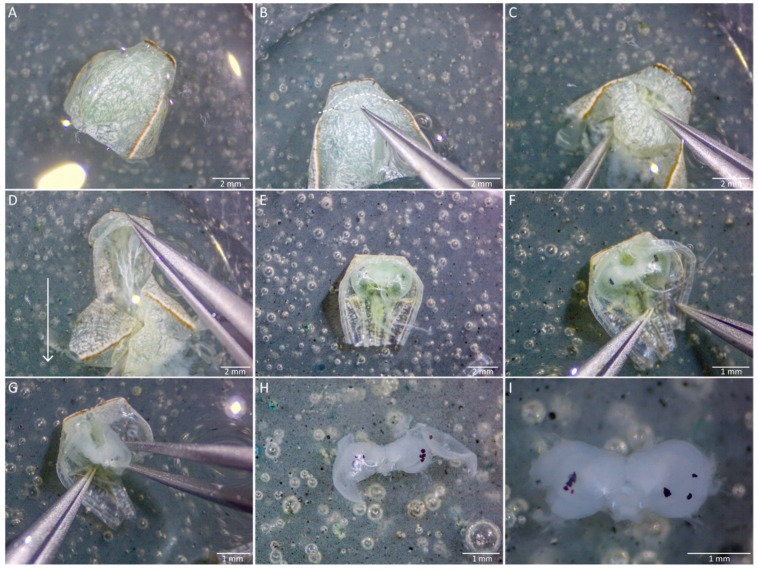
Dissection of a pupal brain at 30% pupal development. (**A**) The anterior part of the pupa is submerged into 1× PBS. (**B**) The thorax cuticle is pushed down and (**C**) detached at the suture at the base of the head (along the dashed line) to make a small opening. (**D**) The thoracic tissue and cuticle are pulled away horizontally from the head tissue (following the direction of the arrow). (**E**) The developing head is visible at the bottom of the pupal cuticle. (**F**) Extra pieces of pupal cuticle are removed. (**G**) Then, the brain is gently scraped out. (**H**) While detached from the pupa cuticle, extra head tissue can be removed from the brain (e.g., cornea, antennae, trachea (seen in a bright white colour)). (**I**) The clean brain can be used for downstream analyses.

- Cut the pupae into two pieces at the base of the thoracic region using the scalpel blade, and move the head and thoracic regions (Figure 2C) into the drop of PBS (Figure 5A). The abdominal part of the cut pupa can be discarded;
- With one hand, hold the cut pupae inside the PBS drop using forceps. With the other hand, use the forceps tips to push the thorax cuticle down at the base of the head (Figure 5B). The junction between the head and the thorax will break, making a small opening in the cuticle (Figure 5C);
- Hold the cuticle head at the opening just made, and with the other hand, grab the thorax cuticle. Pull the thoracic region and all the tissue that come together (antennae, legs for example, Figure 2C) horizontally towards you while firmly holding the head cuticle (Figure 5D). After it is fully detached from the head cuticle, discard the thoracic tissue. The developing butterfly head and the dorsal side of the brain will be exposed at the bottom of the pupal cuticle (Figure 5E);

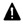
**CRITICAL STEP** Do not pull the thoracic region upward. Pull gently in a horizontal direction to prevent the brain from tearing and being detached from the head capsule;

- Hold the pupal head down in the PBS with one hand, and use your dominant hand to remove remaining thoracic, antennal, and mouthpart tissues still attached to the cuticle and to the developing head (Figure 2C). With your dominant hand, grab the trachea developing around each eye and pull it away;
- Holding the pupal head cuticle down with one hand, use your dominant hand to remove pieces of pupa cuticle around the developing head (Figure 5F), and gently detach the head from the cuticle by pushing it aside gently, or scraping it out of the cuticle (Figure 5G);
- When the developing head is completely detached from the pupa cuticle (Figure 5H), remove the non-brain head tissue, such as the ommatidia and the facets around the visual neuropils, and the developing mouth parts on the other side of the brain. Hold the brain in place with the forceps slightly opened, while pulling the non-brain tissue away with the other hand;
- If the brain (Figure 5I) is used for DNA or RNA extraction, grab it with the forceps and move it to the appropriate reagent (100% ethanol or RNAlater, respectively). If the brains are used for immunostaining, suck the brain up from the PBS with a pipette tip and transfer the brain into the fixative solution. Use a very small pipetting volume, large enough to lift the brain, but small enough to prevent taking an excess of PBS. This method will prevent damage or distortion of the brain tissue.

## 5. Dissection of the Larval Brain and Gnathal Ganglion

### 5.1. Preparation for Dissection

- Place the larvae on ice prior to dissection. After a few minutes, the larvae will become immobile and can be easily picked up with flat forceps;
- Wash the dissecting forceps, blade, and dissection plate with 70% ethanol. If the brains are used for RNA extraction, use RNAzap instead;
- Prepare Eppendorf tubes or well plates filled with the solution appropriate for the subsequent experiment conducted with the dissected brain and place them close to the dissection microscope. For DNA and RNA extraction, prepare tubes with about 300 µL of 100% ethanol and RNAlater, respectively. For immunostaining experiments, place about 500 µL of the fixative solution in the wells;
- Place a drop of cold PBS (about 500 µL, to cover the tissue fully) on the dissection plate.

### 5.2. Dissection Procedure

**Figure 6:**
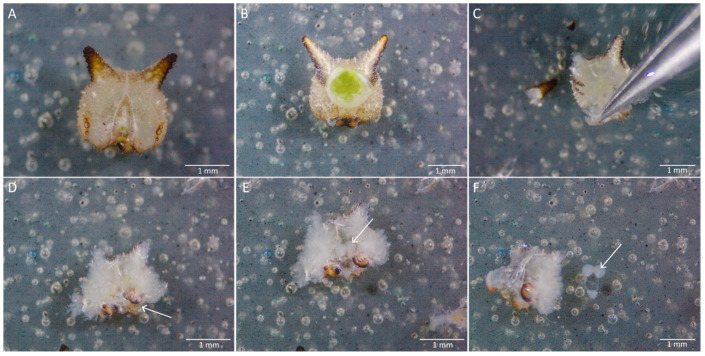
Dissection of the larval brain. (**A**) The head is cut out of the caterpillar body with a scalpel. (**B**) The head is turned face down for an easier dissection. (**C**) Pieces of cuticle are removed piece by piece around the head tissue. (**D**) The mandibles are left attached to the head tissue (arrow), as removing them might damage the brain (**E**). Gently pulling the head tissue on the right and left will create an opening, which exposes the brain (arrow). (**F**) The brain (arrow) attached to the gnathal ganglion (white mass below) can be gently pulled out after removing the pieces of head tissue around it and picked up with a pipette for further downstream experiments.

- Using a scalpel blade, cut the head of the larvae and place it in a drop of PBS on the dissection plate (Figure 6A);
- Place the back of the head towards you and remove the extra thoracic tissue still attached to the hard cuticle (Figure 6B);
- With one hand, hold the head inside the drop of PBS by grabbing the cuticle at the opening. Remove small pieces of the head cuticle using the forceps on your other hand (Figure 6C). The cuticle is hard, so you can break little pieces with the tip of the forceps;

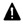
**CRITICAL STEP** Do not pull the mandibles away from the head yet (Figure 2B and Figure 6D). They are attached to the gut, which goes towards the back of the head. passing just above the gnathal ganglion between the connectives (the tissue connecting the lobes and the gnathal ganglion). Pulling the mouthparts would tear the gnathal ganglion and possibly the brain itself, by pulling the connectives away from the brain tissue;

- When most of the back cuticle is removed, turn the head around and proceed to remove the cuticle on the front part of the head. Remove the antennae, the eyes, and other head components attached to the cuticle (Figure 2B). All head soft tissue, including the brain, will remain packed together. When most cuticle is removed, gently scrap the pack of head tissue (with the mandibles still attached) away from the remaining cuticle;
- By gently pulling away some head tissue (by grabbing them from the right and from the left), you can see the two brain lobes and the gnathal ganglion sitting in the middle (Figure 6E). They can be easily recognised from the other tissue, due to their oval shape, smooth texture, and white colour. After identifying the brain, grab and remove pieces of head tissue with one hand, while holding the mandibles with your other hand until the brain and the gnathal ganglion are well exposed. Alternatively, you can grab the group of soft tissue on the right with the corresponding hand, and the other side of the group of tissue with the left hand, and pull them towards opposite directions, freeing the brain placed in between. Pull gently and pay particular attention not to pull the connectives that link the brain with the gnathal ganglion;
- When the brain and the gnathal ganglion are well exposed, remove the other extra tissue still attached to them, by maintaining the brain with one hand through using the forceps slightly opened, and pulling the extra tissue away from it with the other hand. Sometimes, the brain and gnathal ganglion can be extracted directly from the head tissue, by pulling them gently with the forceps (Figure 6F). Small trachea (identified thanks to their shiny white colour) that are strongly attached to each brain lobe should be left in place, as they can tear the brain tissue if pulled away;
- Pick up the brain and the gnathal ganglion using a pipette and transfer them to the reagent appropriate for downstream experiments.

## 6. Expected Results

### 6.1. Adult Brain

**Figure 7:**
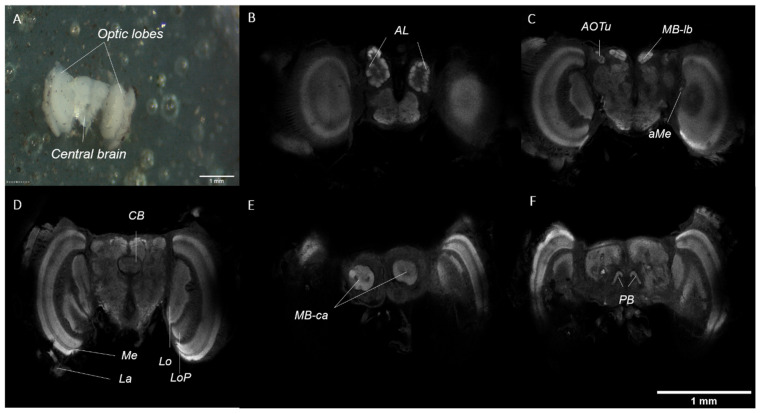
(**A**) Adult brain after dissection, showing the two optic lobes and the central brain (**B**–**F**). Visualisation of the adult brain, immunostained with an antibody against synapsin, in different confocal sections from most anterior (**B**) to most posterior (**F**). (**B**) Antennal lobes (AL). (**C**) Accessory medulla (aMe), anterior optic tubercles (AOTu), and mushroom body lobes (MB-lb). (**D**) Lamina (La), medulla (Me), the lobula (Lo), lobula plate (LoP), and the central body (CB). (**E**) Mushroom body calyx (MB-ca). (**F**) Protocerebral bridge (PB).

The adult brain is made up of two optic lobes and a central mass, which are clearly defined (Figure 7A). A more in-depth visualisation of the brain anatomy can be achieved through immunostaining, using an antibody targeting synapsin (involved in the regulation of neurotransmitter release at synapses) and confocal microscopy techniques. In brief, the optic lobes consist of five paired neuropils, which are clearly defined: the lamina (La), medulla (Me), accessory medulla (aMe), lobula (Lo), and lobula plate (LoP). The central brain consists of five paired and one unpaired neuropils, which are also clearly defined: the antennal lobes (AL), anterior optic tubercles (AOTu), mushroom body lobes (MB-lb) and calyx (MB-ca), protocerebral bridge (PB), and the central body (CB) (Figure 7B–F).

The extraction of a single dissected adult brain using the Rneasy Plus Mini Kit (Qiagen, Singapore) yielded an average of 4500 ng of mRNA, which is a sufficiently large amount for further experiments (e.g., qPCR, RNAseq).

### 6.2. Pupal Brain

**Figure 8:**
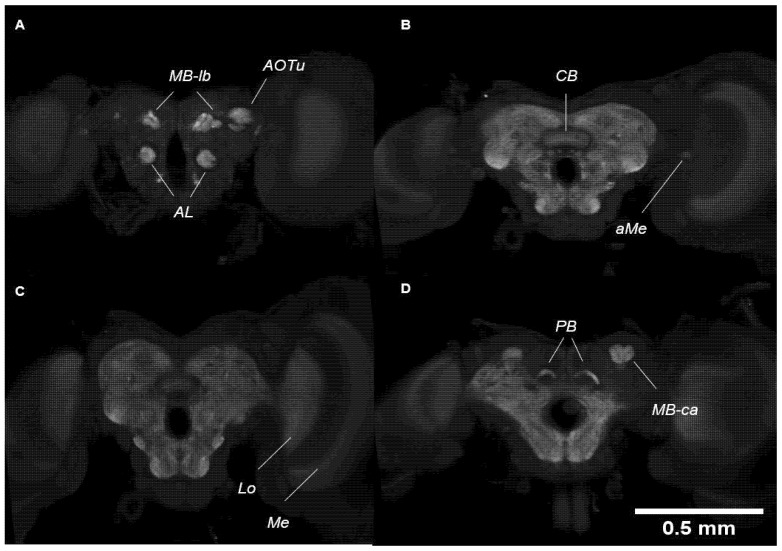
Visualisation of the pupal brain in different confocal sections from most anterior (**A**) to most posterior (**D**) stained with synapsin. (**A**) Mushroom body lobes (MB-lb), anterior optic tubercles (AOTu), and antennal lobes (AL). (**B**) Central body (CB) and accessory medulla (aMe). (**C**) Medulla (Me) and lobula (Lo). (**D**) Mushroom body calyx (MB-ca) and protocerebral bridge (PB).

Similar to the adult brain, the pupal brain also consists of two optic lobes and a central mass which are clearly defined. Visualisation under immunostaining (targeting synapsin) shows three paired neuropils in the optic lobes and five paired and one unpaired neuropil in the central brain. In brief, the neuropils visualised in the optic lobes are the medulla (Me), accessory medulla (aMe), and the lobula (Lo) (Figure 8B,C). In the central brain, the paired neuropils are the antennal lobes (AL), anterior optic tubercles (AOTu), mushroom body lobes (MB-lb) and calyx (MB-ca), protocerebral bridge (PB), and the central body (CB) (Figure 8A,B,D). However, the AL and the MB-ca are not as well-defined or well-developed in the pupal brain than as in the adult brain. 

The extraction of a single dissected pupal brain using the Rneasy Plus Mini Kit (Qiagen, Singapore) yielded an average of 3000 ng of mRNA, a sufficiently large amount that can be used for further experiments (e.g., qPCR, RNAseq).

### 6.3. Larval Brain

**Figure 9:**
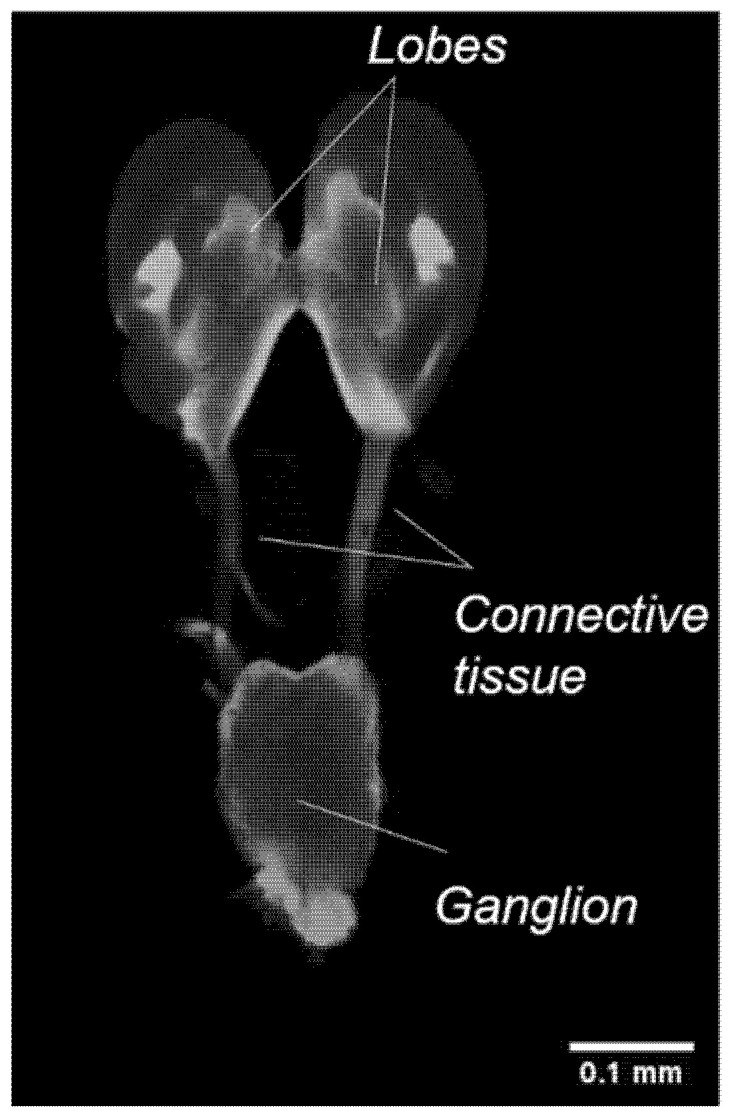
Visualisation of the larval brain, immunostained with an antibody against synapsin in a single confocal section, showing the lobes, connective tissues, and ganglion.

The larval brain appears relatively different from the adult and pupal brains. The larval brain consists of two fused lobes connected dorsally to a ventral ganglion by connective tissues. Further analysis of the brain by immunostaining did not reveal any defined neuropils (Figure 9).

The extraction of a single dissected larval brain using the Rneasy Plus Mini Kit (Qiagen, Singapore) yielded an average of 1000 ng of mRNA. This amount is sufficient for subsequent qPCR analysis. Several larval brains can be pooled into one tube, to get a higher RNA yield as required by some sequencing companies.

### 6.4. Brains of Other Butterfly Species

**Figure 10:**
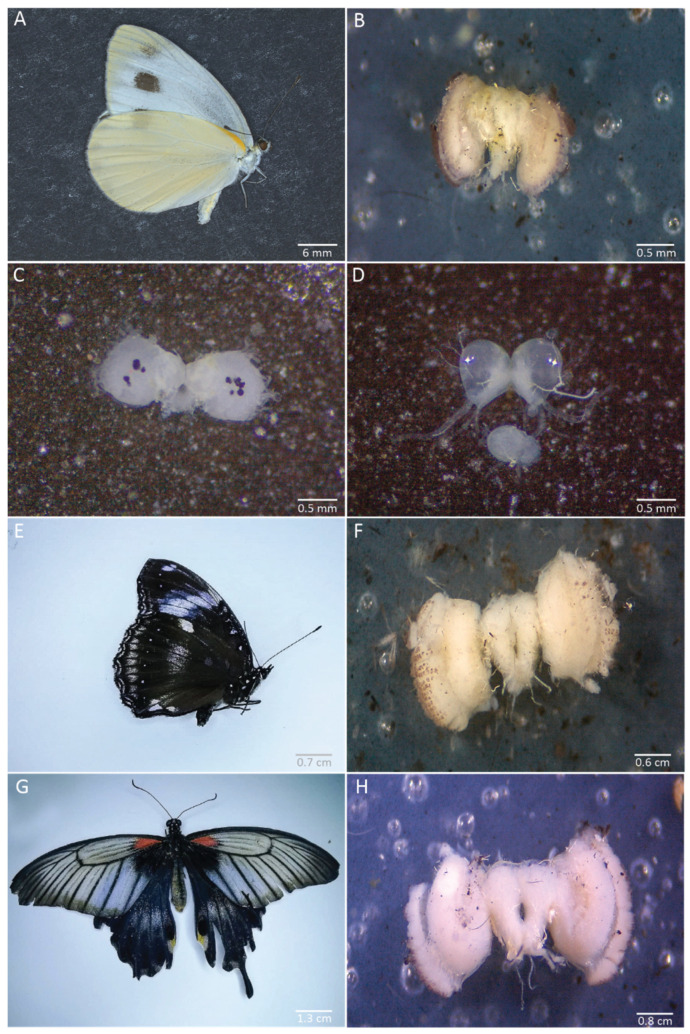
Brains dissected from *Pieris canidia, Hypolimnas anomala,* and *Papilio sp.* (**A**–**C**) *Pieris canidia* brain at the adult stage (fixed brain, **B**), at 30% pupal development (not fixed, **C**), and the 5th instar larval stage (not fixed, **D**). (**F**) Adult brain from *Hypolimnas anomala* (Nymphalidae, **E**). (**H**) Adult brain from *Papilio sp* (Papilionidae, **G**).

We applied our dissection procedure to brains of other species from different families and of various sizes. We successfully dissected the brain of *Pieris canidia* at the same stages as in *B. anynana:* adult, 30% pupal development, and 5th instar larva (Figure 10A–D). We also successfully dissected the brains of Malayan eggfly adults (Nymphalidae) (Figure 10E,F) and of *Papilio sp* butterflies (Papilionidae) (Figure 10G,H).

## 7. Reagents

### 7.1. Preparation of HEPES-Buffered Saline (HBS)

- Add 800 mL of MilliQ water and reagents mentioned in Table 1 to a 1 L glass bottle;
- Measure the pH of the solution using a pH metre, and adjust accordingly with drops of HCl and NaOH until the solution has a pH of 7.4;
- Then, add MilliQ water until the volume in the bottle reaches 1 L;
- Autoclave the solution and store it at 4 °C thereafter.

### 7.2. Preparation of 10× Phosphate-Buffered Saline (PBS)

- Add 800 mL of MilliQ water and reagents mentioned in Table 2 to a 1 L glass bottle;
- Measure the pH of the solution using a pH metre and adjust accordingly with drops of HCl and NaOH until the pH is around 6.8;
- Add MilliQ water until the volume in the bottle reaches 1 L;
- Autoclave the solution and store it at 4 °C thereafter;
- For the preparation of 100 mL of 1× PBS, dilute 10 mL of 10× PBS into 90 mL of MilliQ water.

### 7.3. Preparation of Zinc Formaldehyde (ZnFA)

- Add 800 mL of MilliQ water and reagents mentioned in Table 3 to a 1 L glass bottle;
- Measure the pH of the solution using a pH metre and adjust accordingly with drops of HCl and NaOH until the pH is 6.35;
- Add MilliQ water until the volume in the bottle reaches 1 L and autoclave the solution for 20 min at 121 °C;
- Add 4% Formaldehyde and store it at 4 °C;
- For the preparation of 4% Formaldehyde, dilute 10 mL of 16% Formaldehyde in 30 mL of 1× PBS.

## Figures and Tables

**Table 1:** Reagents used to make 1 L of HBS.

Reagents	Amount (g)
NaCl	8.77
KCl	3.73
CaCl_2_	5.55
Sucrose	8.56
HEPES	2.38

**Table 2:** Reagents used to make 1 L of 10× PBS.

Reagents	Amount (g)
NaCl	81.80
KH_2_PO_4_	5.28
K_2_HPO_4_	10.68

**Table 3:** Reagents used to make 1 L of ZnFA.

Reagents	Amount
ZnCl	2.51 g
NaCl	7.89 g
Sucrose	11.98 g
4% Formaldehyde	10 mL

## Data Availability

All the data collected during this study are available in the article.
